# The Catalytic Reduction of Nitroanilines Using Synthesized CuFe_2_O_4_ Nanoparticles in an Aqueous Medium

**DOI:** 10.1002/open.202200156

**Published:** 2022-11-03

**Authors:** Samin Naghash‐Hamed, Nasser Arsalani, Seyed Borhan Mousavi

**Affiliations:** ^1^ Research Laboratory of Polymer Department of Organic and Biochemistry Faculty of Chemistry University of Tabriz Tabriz Iran; ^2^ J. Mike Walker ‘66 Mechanical Engineering Department Texas A&M University College Station TX 77843 USA

**Keywords:** catalysis, magnetic nanoparticles, nitroaniline, reduction, wastewater treatment

## Abstract

The primary objective of this research is to investigate the reduction of 4‐nitroaniline (4‐NA) and 2‐nitroaniline (2‐NA) using synthesized copper ferrite nanoparticles (NPs) via facile one‐step hydrothermal method as a heterogeneous nano‐catalyst. Nitroanilines were reduced in the presence and without the catalyst with a constant amount (100 mg) of reducing agent of sodium borohydride (NaBH_4_) at room temperature in water to amino compounds. To characterize the functional groups, size, structure, and morphology of as‐prepared magnetic NPs, FTIR, XRD, SEM, and TEM were employed. The UV‐Vis spectrum was utilized to explore the catalytic effect of CuFe_2_O_4_. The outcomes revealed that the synthesized CuFe_2_O_4_ as a heterogeneous magnetic nano‐catalyst catalyzed the reduction of 4‐NA and 2‐NA significantly faster than other candidate catalysts. The outcomes demonstrated that the catalyst catalyzed 4‐nitroaniline to *para*‐phenylenediamine (*p*‐PDA) and 2‐nitroaniline to *ortho*‐phenylenediamine (o‐PDA) with a constant rate of 7.49×10^−2^ s^−1^ and 3.19×10^−2^ s^−1^, and conversion percentage of 96.5 and 95.6, in 40 s and 90 s, sequentially. Furthermore, the nanoparticles could be recovered by a magnetic separation method and reused for six consecutive cycles without remarkable loss of catalytic ability.

## Graphical abstract

**Magnetic CuFe_2_O_4_ nanoparticles** were synthesized as an effective, economical, and environmentally friendly nano‐catalyst for reducing 4‐ and 2‐nitroaniline using sodium borohydride as reducing agent. The heterogeneous nano‐catalyst was synthesized using a facile one‐step hydrothermal method.

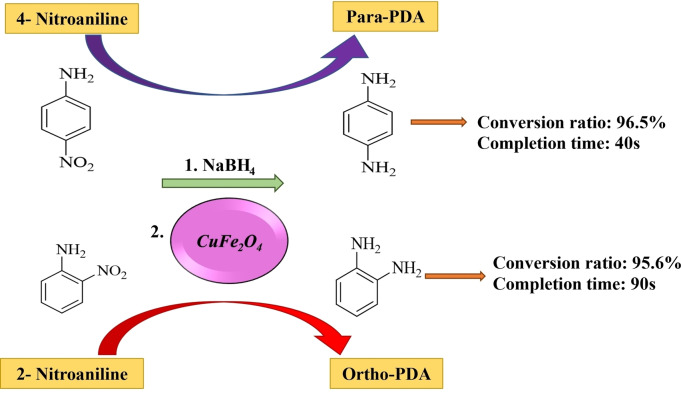

## Introduction

Water contamination and environmental pollution are among the crucial issues in today's advanced world.[^1^, ^2^] Nitroaniline (NA) is a significant aromatic amine commonly used as a precursor and an intermediate in the organic synthesis of products such as oxidizing agents, drugs, dyes, and pesticides.[^3^, ^4^] Nitroaromatic compounds have a detrimental impact on human health and the environment, such as nitroarenes, which can result in many diseases like anemia and lung injury through breathing, feeding, and absorption through the skin.^[5]^ Nitroanilines as a pollutant have been listed by several countries that need careful control due to their difficulty in the degradation process.^[6]^ Various techniques have been employed for water treatment purposes, including physical, chemical, and biological.[^7^, ^8^] The physical techniques cannot completely remove pollution, thus, resulting in further contamination.^[9]^ The benefit of using chemical reduction of nitroaromatic compounds to amino compounds is the production of non‐toxic or low‐toxic amino compounds. In addition, amino compounds are used as a raw material in the preparation of medicines; for example, the reduction product of 2‐nitroaniline is *ortho*‐phenylenediamine (*o*‐PDA).^[10]^ Nowadays, one of the most common ways to reduce nitroaromatic compounds is utilizing nano‐catalysts.^[11]^

Nanoparticles (NPs) have been used in many applications due to their remarkable properties, such as large surface area, high heat capacity, structural stability, low melting temperature, high surface energy, and high diffusion.[^12^, ^13^, ^14^, ^15^, ^16^, ^17^, ^18^] CuFe_2_O_4_ NPs, such as spinels of the MFe_2_O_4_ (M=Cu, Co, Mn, Zn, *etc*.) kind possess prominent electrical and magnetic properties.^[19]^ Compared with other nano‐catalysts, copper ferrite has many advantages, such as biocompatibility, high magnetic property, thermal firmness, electronic conductivity, and productive catalytic activity.^[20]^ The magnetic catalysts can be easily removed in the water treatment step, preventing catalyst loss and making the catalyst economically viable.^[21]^ Copper ferrite NPs can be obtained via various methods, including hydrothermal, solvothermal, ball milling, microwave, ultra‐sonication, sol‐gel, and co‐precipitation.[^22^, ^23^, ^24^, ^25^] Among the wide range of magnetic materials, CuFe_2_O_4_ magnetic NPs can be easily prepared via the hydrothermal method, which are recyclable and show good particle dispersion characteristics.^[21]^

Farooqi et al.^[26]^ prepared an efficient catalyst by fabricating the Ag NPs into the p (NiPA‐co‐AAc) microgels to reduce 4‐NA at various concentrations. They found that the rate constant of nanoparticles increased with increasing the concentration of catalyst and reducing agent. Das et al.^[27]^ analyzed the impact of nanocomposites on reducing nitro compounds resulting from dyes in wastewater. The results exhibited that the synthesized Ni−RGO nanocomposites via the hydrothermal method could remove the dye concentration by about 93 % at 190 min. Moreover, the prepared nanocomposites had better reduction activity of 4‐NA compared to the Ni NPs and RGO. Sadeghzadeh et al.^[28]^ evaluated the magnetic nano‐catalyst of UiO‐66‐NH_2_/TTACP/Ni@Pd to reduce 2‐nitroaniline. They reported that 1 mg of the prepared nano‐catalyst in a reaction could reduce the 2‐nitroaniline at least 10 times with a *k_app_* of 1.42×10^−2^ s^−1^ in 150 s. Bhaduri et al.^[29]^ conducted a study to investigate the effect of (Au/SiO_2_/Fe_3_O_4_) composite as a catalyst on the reduction of 2‐nitroaniline (2‐NA). The outcomes demonstrated that gold nanocomposites remarkably reduced the 2‐NA with a conversion rate, time, and constant rate of 100 %, 225 s, 4.1×10^−3^ s^−1^, respectively. The catalytic performance of CuFe_2_O_4_/RGO composites was assessed by Othman et al.^[30]^ in the reduction of phenol. They found that the synthesized composites had a remarkable capability in reducing phenol compounds and eliminating organic compounds from aqueous solutions. They also asserted that the prepared CuFe_2_O_4_/RGO composites were considerably better than expensive TiO_2_ NPs. Amulya et al.^[31]^ investigated the impact of CuFe_2_O_4_ NPs as a photocatalyst on eliminating dyes. The results showed that CuFe_2_O_4_ NPs could remove the methylene blue (MB) and drimarene yellow (DY) dyes and showed their photocatalytic effect in the degradation of pigments. CuFe_2_O_4_‐CDs nanocomposites were fabricated as a reusable catalyst by Wang et al.^[32]^ The influence of CuFe_2_O_4_/CQD nanocomposites on removing organic pollution was investigated and the organic nitroarene (4‐nitrophenol) was removed from water pollution using the catalyst. Arumugam et al.^[33]^ conducted a study to reduce various dyes (methylene blue (MB) and Allura red (AR)) along with nitroarene compounds (2‐nitroaniline, 3‐nitroaniline, 4‐nitroaniline, and 4‐nitro‐2‐phenylenediamine (4‐NPDA) with a synthesized TCPIL/CuFe_2_O_4_/BNONS nanomaterial as a nano‐catalyst. The outcomes showed that 4‐nitro‐2‐phenylenediamine (4‐NPDA) was reduced faster than other compounds. Furthermore, increasing the catalyst amount and temperature efficiently impacted the reduction process of compounds and dyes. The catalytic effect of CuFe_2_O_4_ magnetic NPs and the reduction of nitrobenzene to aniline and benzonitrile to benzylamine were studied by Zeynizadeh et al.^[34]^ The results showed that the fabricated copper ferrite could reduce the nitrobenzene in 50 min with a constant rate of 8.32×10^−2^ min^−1^.

According to the literature review, most research studies in the reduction field have focused on reducing nitrophenol compounds or degrading dyes in the wastewater; however, no research has been conducted on reducing nitroaniline compounds via catalytic and photocatalytic methods employing multi‐step or non‐economical synthesis methods.[^25^, ^35^] Moreover, no studies have been conducted on nanoparticles′ facile and convenient synthesis methods and using sufficient precursors to synthesize a suitable catalyst. Moreover, the morphology of the synthesized nanoparticles was either spherical or cubic;^[—35,36]^ however, the synthesized nanoparticles in this study were in a combination form. In this study, CuFe_2_O_4_ magnetic NPs with different morphologies in terms of low‐cost raw substances, facile synthetic method, easy to separate from the aqueous medium, fast, and recyclable catalyst were synthesized.

Herein, for the first time, we fabricated the magnetic CuFe_2_O_4_ NPs via a one‐step hydrothermal method that, followingly, demonstrated a fast rate in a reduction reaction of nitroarenes (4‐nitroaniline and 2‐nitroaniline) at room temperature. The catalytic efficiency of obtained NPs was investigated in reducing nitroanilines with a reducing agent of sodium borohydride via UV‐Vis spectroscopy. The catalytic reusability of the synthesized nanoparticles was studied for up to at least six cycles.

## Experimental Section

### Materials

Iron(III) chloride (FeCl_3_), copper(II) chloride dihydrate (CuCl_2_ ⋅ 2H_2_O), ethanol (C_2_H_5_OH), dodium hydroxide (NaOH), dodium borohydride (NaBH_4_), 2‐NA (C_6_H_6_N_2_O_2_), and 4‐NA (C_6_H_6_N_2_O_2_) were commercially purchased from Merck, Germany. Ethylenediaminetetraacetic acid tetrasodium salt (EDTA) (C_10_H_14_N_2_ Na_4_O_8_) was purchased from Sigma‐Aldrich.

Fourier transform infrared spectroscopy (FTIR, Bruker, TENSOR 27, Germany) was employed to characterize the functional groups. To assess the morphology and size of nanoparticles, a scanning electron microscope (SEM, Carl Zeiss 1430VP L, Germany) and transmission electron microscopy (TEM, EM 208S, Philips, Netherlands) at a voltage of 100 kV were used. To determine the approximate size and crystallite structure of nanoparticles, X‐ray diffraction (XRD, PW1730, Philips, Netherlands) (Cu−Kα (*λ*=1.54056 A°) radiation at a voltage of 40 kV and 30 mA was applied. The XRD patterns were scanned over the angular radius of 2*θ* = 10–80° with a step size of 0.05° and time per step of 1 s. An analysis of UV‐visible absorption spectra (Specord 250, Analytik Jena) was carried out to exhibit the catalytic properties of nanoparticles. A pH meter (AZ 86502, Iran) was used to regulate the exact pH number of solutions. In order to prevent the agglomeration of solutions, the magnetic stirrer (Fan Azma Gostar, Iran) was utilized. A heating oven (Binder, ED 23) was employed to obtain the magnetic nanoparticles.

### Synthesis of CuFe_2_O_4_ nanoparticles

**Figure 1:**
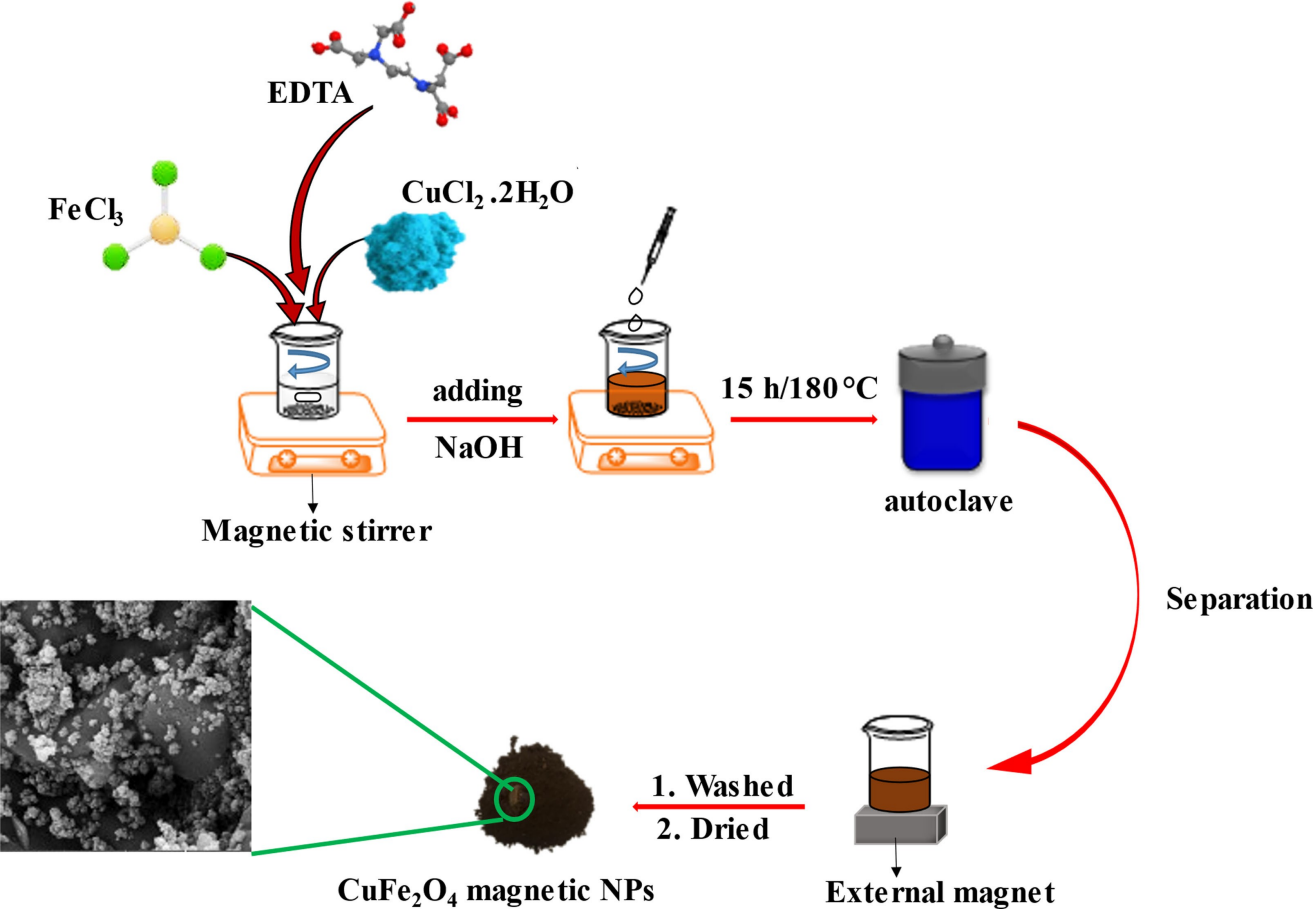
Synthesis of brownish copper ferrite NPs by one‐step hydrothermal method.

The CuFe_2_O_4_ NPs were synthesized via a one‐step hydrothermal method. Figure 1 demonstrates the schematic of the preparation procedure. First, 0.574 g (1.509 mmol) of EDTA tetrasodium salt as a chelator was dispersed in 50 mL deionized water (DI) in the ultrasonic cleaner bath for 10 min. Then, the stoichiometric amount of 3.24 g (19.97 mmol) of FeCl_3_ and 1.35 g (7.918 mmol) of CuCl_2_ ⋅ 2H_2_O were added to the solution under continuous stirring. The NaOH solution, prepared by adding 17.7 g (0.442 mol) per 100 mL of water, was poured into the solution with a dripping syringe (during the reaction, the pH of the reaction was constantly monitored by a pH meter); when the pH was reached 10–11, the solution was transferred into the stainless steel Teflon autoclave and reacted for about 15 h at 180 °C. Afterward, a robust external magnet allowed for magnetic precipitate separation. The precipitate was washed with ethanol and distilled water several times. Finally, the prepared precipitate was dried at 70 °C for about 3 h.

### Catalytic evaluation

The catalytic reduction stages for CuFe_2_O_4_ and in the absence of any catalyst were accomplished in a quartz cuvette. In order to pursue the progress of reduction of 4‐NA and 2‐NA as an example of organic compounds in the existence of NaBH_4_, UV‐Vis spectrophotometer was conducted. 10 mg of each nitroaromatic was dissolved in 50 mL of DI water for 15 min to perform the experiment. Then, 5 mL of the prepared solution was poured into the testing tube, 100 mg of NaBH_4_ as a reducer, and 3.5 mg of catalyst were added. Finally, 100 μL of the produced solution was transferred rapidly into the cuvette, and its absorption was taken once a few seconds until the color of the solution wholly changed.

## Results and Discussion

### Characterization

**Figure 2:**
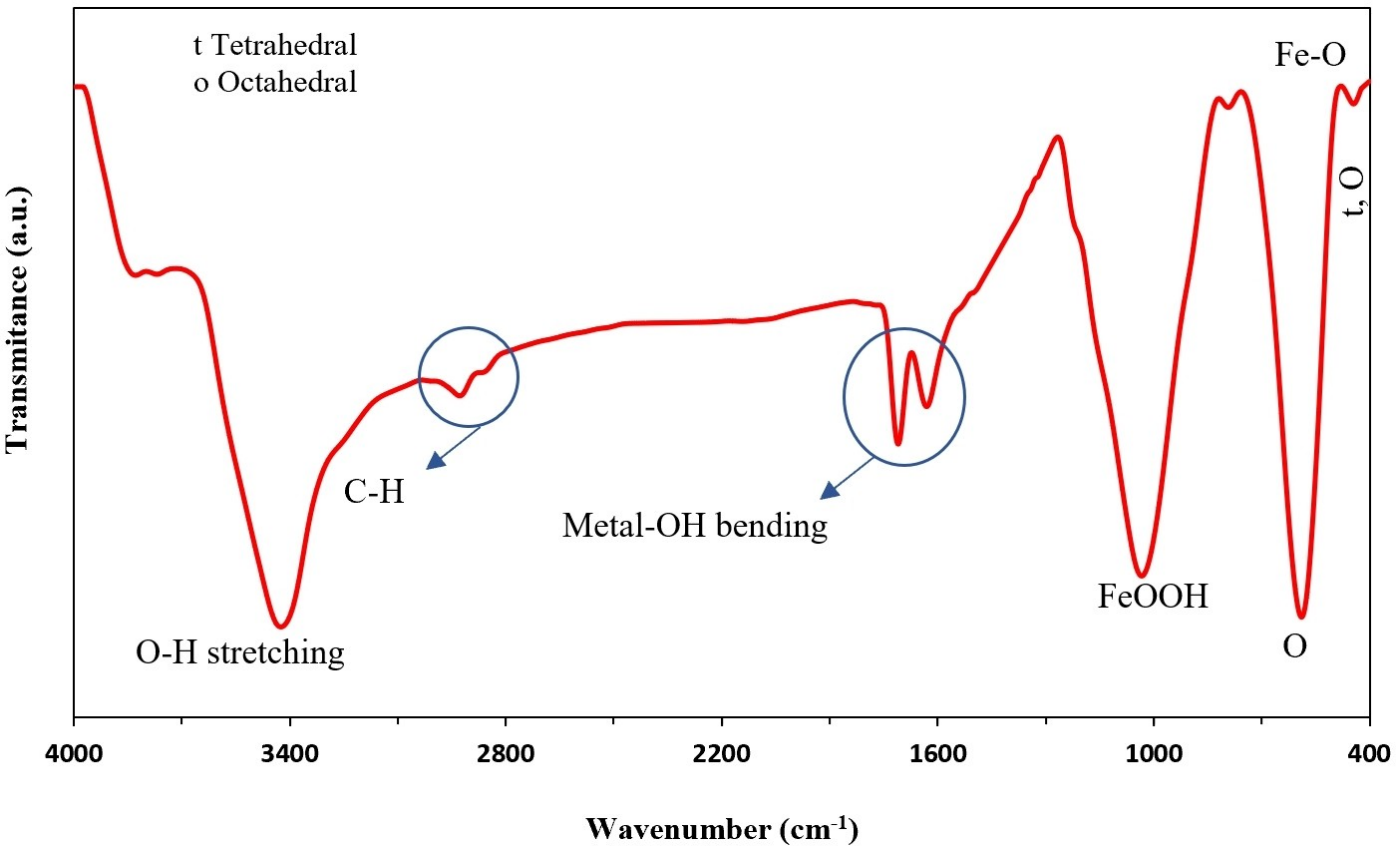
FTIR of CuFe_2_O_4_ NPs.

Fourier‐transform infrared spectroscopy was employed to investigate the various functional groups of as‐synthesized CuFe_2_O_4_ NPs. Figure 2 displays the FTIR of CuFe_2_O_4_ NPs. The high‐frequency band ∼594 cm^−1^ refers to the deformation of Fe−O in octahedral and tetrahedral sites. In comparison, the low‐frequency ∼451 cm^−1^ is attributed to the deformation of Fe−O in the octahedral hematite site. The peak seen around 2900 cm^−1^ corresponds to the C−H band. The bond of FeOOH was seen at 1035 cm^−1^. The O−H group stretching vibration in the water on the surface of NPs is seen in the 3500 cm^−1^. The two bonds, seen at 1627 cm^−1^ and 1700 cm^−1^, correspond to a metal−OH (hydroxyl group) bond bending vibration.[^31^, ^37^, ^38^, ^39^]

**Figure 3:**
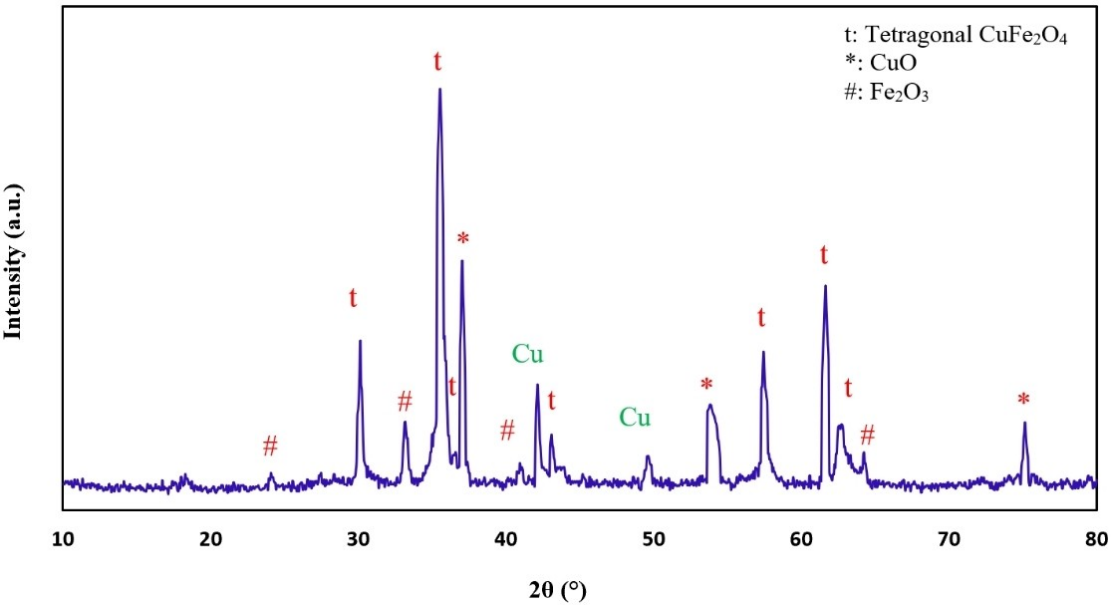
XRD patterns of CuFe_2_O_4_ NPs.

The XRD pattern of CuFe_2_O_4_ NPs is shown in Figure 3. As can be seen, the X‐ray diffraction for the prepared sample has seven major strong peaks at 2*θ*=30.2°, 35.6°, 36.64°, 43.14°, 57.4°, 61.64°, and 62.84° corresponded to (112), (211), (202), (220), (312), (224), (400), sequentially. This sample‘s diffraction is in a good acceptance of the JCPDS card (34‐0425), which depicts the tetragonal shape of copper ferrite NPs.^[40]^ A strong peak at 2*θ*=42.09° is indexed to Cu.^[41]^ Furthermore, prominent peaks are seen at 2*θ*=37.4° (*111), 49.5° (*−202), 53.94° (*020), 75.09° (*220), corresponding to the monoclinic CuO phase plane (JCPDS No. 80–1916).[^42^, ^43^] In terms of Fe^3+^ and Cu^2+^ complex constants, the presence of CuO may be reasonable. The complex constant of Fe^3+^ (logβ=20.19±0.02) is larger than that of Cu^2+^ (logβ=8.08), so Fe^3+^ complexes should be more stable than Cu^2+^. Therefore, Cu^2+^ agglomerates with NaOH and finally forms CuO. Some secondary impurities, such as Fe_2_O_3_ were still found, which may be attributed to the insolubility of FeO, in a good acceptance of the JCPDS card (33‐0664).[^31^, ^41^]

The Debye–Scherrer equation was utilized to calculate the crystallite size of copper ferrite NPs, based on the x‐ray diffraction pattern.^[45]^

(1)

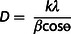




where *D*, *λ*, *β*, *θ*, and *k* are assigned to the mean crystallite size of nanoparticles (nm), the X‐ray wavelength, the line broadening at the half summit of the maximum of the XRD diffraction (FWHM), the Bragg angle, and shape factor which is about 0.9. The crystalline size of the copper ferrite NPs, which correspond to the (211), was calculated by Equation (1) and their average size was found at 42.3 nm. Overall, the sharp peaks indicate the crystalline nature of synthesis products, and no impurity peaks were identified in the pattern.^[46]^

**Figure 4:**
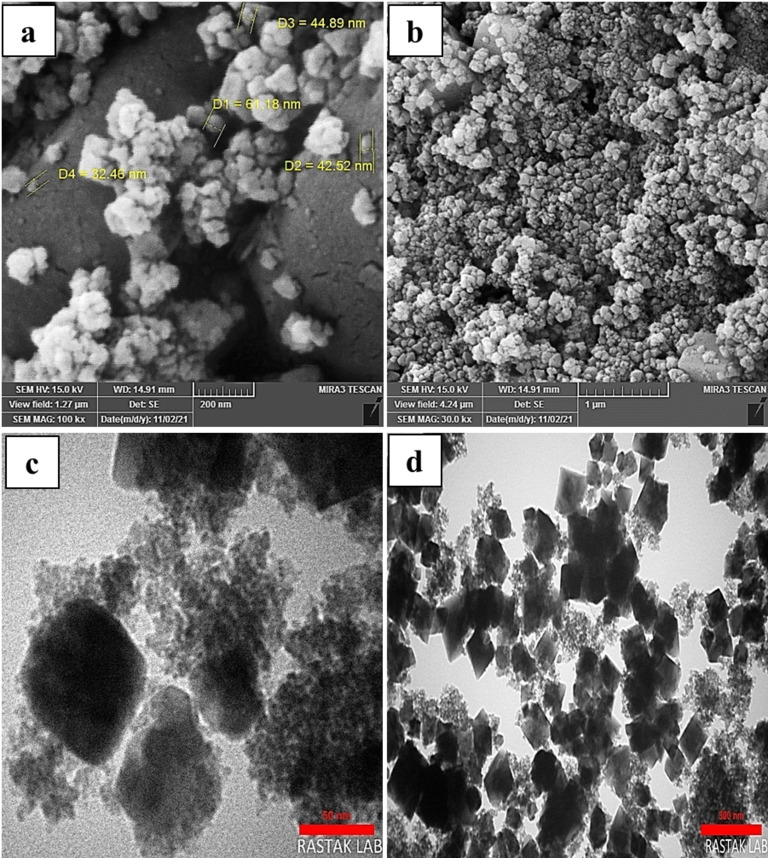
(a, b) SEM images and (c, d) TEM images of CuFe_2_O_4_ NPs.

SEM and TEM monitored the morphology images of the synthesized CuFe_2_O_4_ NPs. SEM analysis was employed to investigate the surface morphology of nanoparticles. Also, TEM analysis was carried out to get more insight into the nanoparticles and accurate information about their structure and crystallite. Figures 4(a, b) show the SEM and Figures 4(c, d) show the TEM pictures of the CuFe_2_O_4_ NPs. The magnetic properties of CuFe_2_O_4_ NPs led to agglomeration.^[47]^ Spherical and cubic shapes are recognized in the morphology of the nanoparticles because over half of the particles were prepared when the reaction was mixed under a magnetic stirrer (before the solution was poured into the stainless autoclave). In contrast, other nanoparticles, which did not have the opportunity to grow, grew up in the heating oven via a hydrothermal reaction.

**Figure 5:**
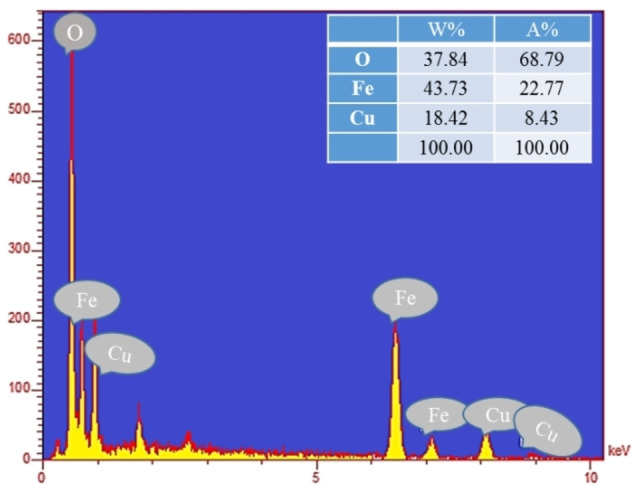
The EDS spectrum of the CuFe_2_O_4_ NPs.

**Figure 6:**
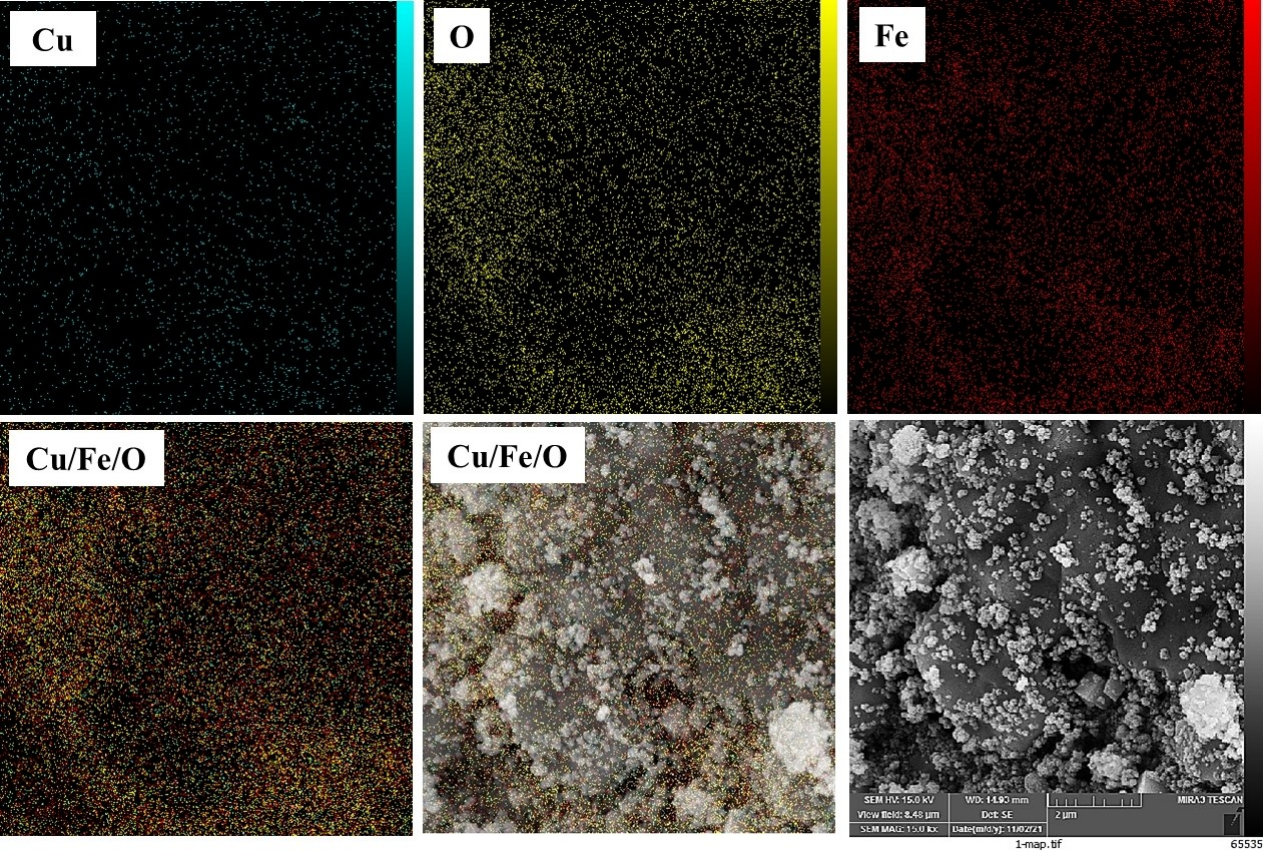
EDS mapping images of the CuFe_2_O_4_ NPs.

The EDS spectrum of the CuFe_2_O_4_ NPs is shown in Figure 5. In Figure 5, the presence of Cu, Fe, and O elements in the NPs structure is proved. According to the table of quantitative results, it is observed that the atomic ratio of Fe to Cu is about 2.50, which can confirm the dosage ratio of Fe/Cu (2.00) in the formula of the copper ferrite. The EDS mapping pictures of the CuFe_2_O_4_ NPs are presented in Figure 6. Clearly, three main elements, including Fe, Cu, and O, are seen. EDS images also showed a higher molar ratio of O rather than other elements, which confirmed the proper synthesis of NPs. As clear in Figure 4(a), CuFe_2_O_4_ NPs have an average diameter size of about 45 nm, demonstrating a good agreement with 2*θ*=35.6° corresponding to the (211) in the XRD patterns.

Experimental outcomes provided detailed information on the geometry of ferrites of CuFe_2_O_4_ that can be obtained by various methods mentioned in the literature review. Fe(OH)_3_ and Cu(OH)_2_ will be produced as intermediate products when Cu^2+^ and Fe^3+^ ions react in the basic medium. When heating at high temperatures, the hydroxides produce copper ferrite NPs through the following reactions (Eq. (2)–4):

(2) $$\mathrm{Cu}{}^{2+}+2\mathrm{OH}{}^{2-}\to \mathrm{Cu}\;\left(\mathrm{OH}\right){}_{2}$$

(3) $$\mathrm{Fe}{}^{3+}+3\mathrm{OH}{}^{2-}\to \mathrm{Fe}\;\left(\mathrm{OH}\right){}_{3}$$

(4) $$\mathrm{Cu}\;\left(\mathrm{OH}\right){}_{2}+2\mathrm{Fe}\;\left(\mathrm{OH}\right){}_{3}\to \mathrm{CuFe}{}_{2}O{}_{4}+4H{}_{2}O$$

The shape of CuFe_2_O_4_ NPs depends on the treatment temperature, preparation method, and the absence/presence of anhydrous solvents. In addition, the size of magnetic NPs depends on the Cu(OH)_2_ and Fe(OH)_3_ nucleus formation, and the growth rate can be controlled by annealing temperature and solvent.^[48]^

### Catalytic study

**Figure 7:**
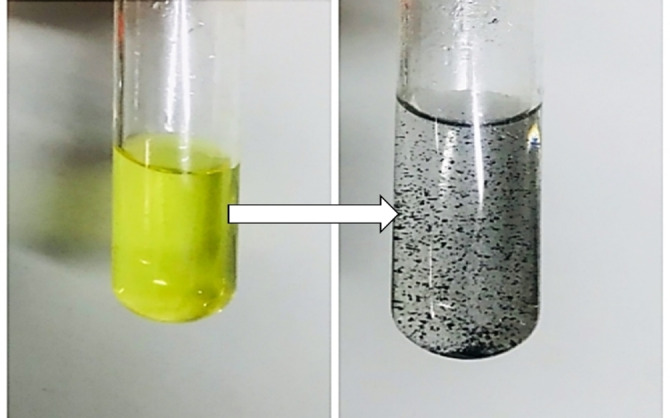
The color transformation of 4‐NA after adding the nano‐catalyst.

The catalytic reduction of 4‐NA to *p*‐PDA (*para*‐phenylenediamine) and 2‐NA to *o*‐PDA (*ortho*‐phenylenediamine) was investigated using CuFe_2_O_4_ NPs as a heterogeneous catalyst. The reduction of NA was monitored via UV‐Vis absorption spectroscopy in the wavelength range of 250–550 nm. To study the effect of the nano‐catalyst on the 4‐NA and 2‐NA as a model of reduction in the aqueous solution, a constant amount of catalyst (3.50 mg) was added after adding NaBH_4_ as a reducing agent. Then, the reduction progress was monitored by calculating the decrease in absorbance by passing the time. Figure 7 shows that for 4‐NA, the color changed from bright yellow to light grey (colorless) after adding a magnetic nano‐catalyst.

**Figure 8:**
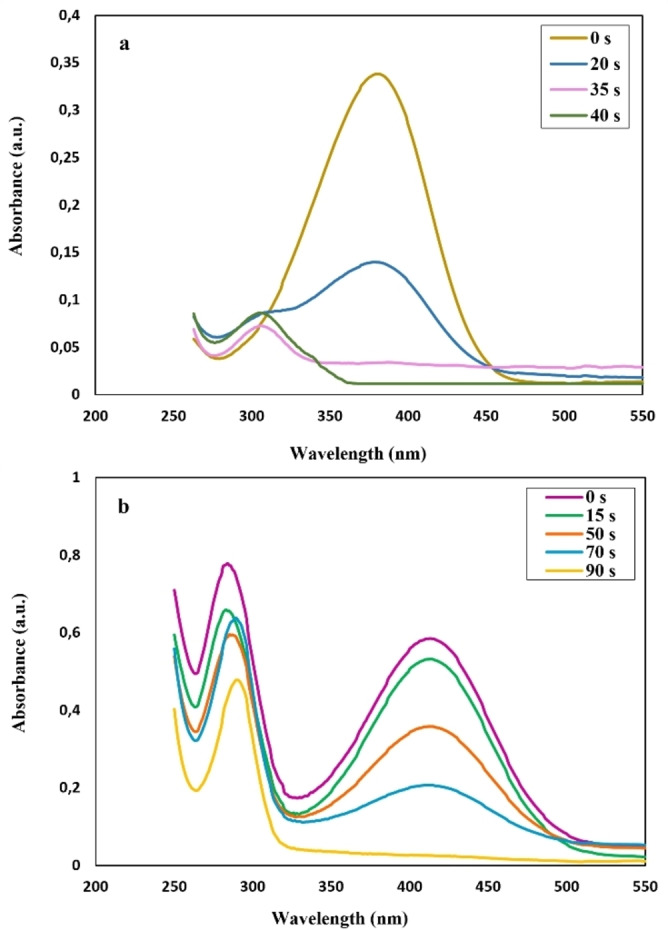
Chemical mechanism of 2‐NA reduction reaction catalyzed by copper ferrite nano‐catalyst.

As shown in Figure 8, the NaBH_4_ as an electron donor and 4‐NA as a substrate become adsorbed on the exterior of the nano‐catalyst; hence, the metal nano‐catalyst is charged and persuaded the hydrogenation of the 4‐NA and 2‐NA. Accordingly, the nitroarenes are reduced to the nitroso compounds; afterward, they are instantly transformed into the hydroxylamine compound. Finally, the corresponding hydroxylamine compound rapidly is changed to the aromatic amine compound.^[49]^

**Figure 9:**
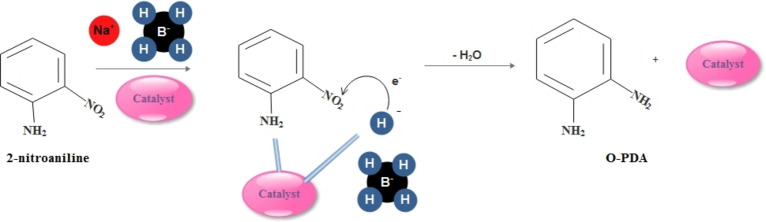
UV‐Vis absorbance of (c) reduction of 4‐NA and (d) reduction of 2‐NA with the addition of nano‐catalyst at room temperature (25 °C) using NaBH_4_ as hydrogen source (reducing agent).

In Figure 9(a), after adding nano‐catalyst, the decline in UV‐Vis absorption at approximately 380 nm and the rising in 307 nm was recognized, owing to the decrease in the concentration of 4‐NA and increasing the concentration of *p*‐PDA at 307 nm.^[26]^ The two prominent peaks at 285 and 415 nm are related to the 2‐NA (Figure 9(b)).^[29]^ In Figure 9(b), with the addition of the nano‐catalyst, absorption of 2‐NA decreased to 415 nm since the concentration of 2‐NA decreased in an aqueous solution. Also, the apparent shift from 285 nm to 290 nm can be seen due to the formation of *o*‐PDA.^[50]^ It can be regarded in Figure 9(a) that when the CuFe_2_O_4_ NPs were added into the solution as a catalyst, absorbance was decreased over 40 s from 0.33 to 0.011 at 380 nm. Moreover, the peak from 0.075 to 0.092 in 307 nm is ascribed to the new product, *p*‐PDA. In Figure 9(b), following the augment of the as‐obtained nano‐catalyst, absorption of 2‐NA decreased to 415 nm due to the diminishing concentration of 2‐NA. The decrease from 0.58 to 0.021 at around 415 nm over 90 s showed the reduction reaction of 2‐NA.

### Studying the reduction ratio and rate of reaction

The conversion ratio of nano‐catalyst to reduce the 4‐NA and 2‐NA catalyzed by magnetic NPs was calculated by Equation 5.^[51]^

(5) $$\mathrm{Conversion}\;\left(\%\right)=\frac{A_{0}-A_{t}}{A_{0}}\times 100$$

where $A_{0}$ and $A_{t}$ are the absorbance of 4‐NA/2‐NA as a substrate in the initial time t=0 and at any time of *t*, respectively.

It can be noted that the concentration of NaBH_4_ is significantly higher than the amount of catalyst. Hence, the reaction rate relies on only the concentration of stated nitroaromatic compounds. Therefore, the reaction follows pseudo first‐order kinetics. In order to estimate the catalytic reduction of compounds, Equation 6 was used.^[27]^

(6) $$\ln\left(\frac{C_{t}}{C_{0}}\right)=\ln\left(\frac{A_{t}}{A_{0}}\right)=-K_{app}\cdot t$$

**Figure 10:**
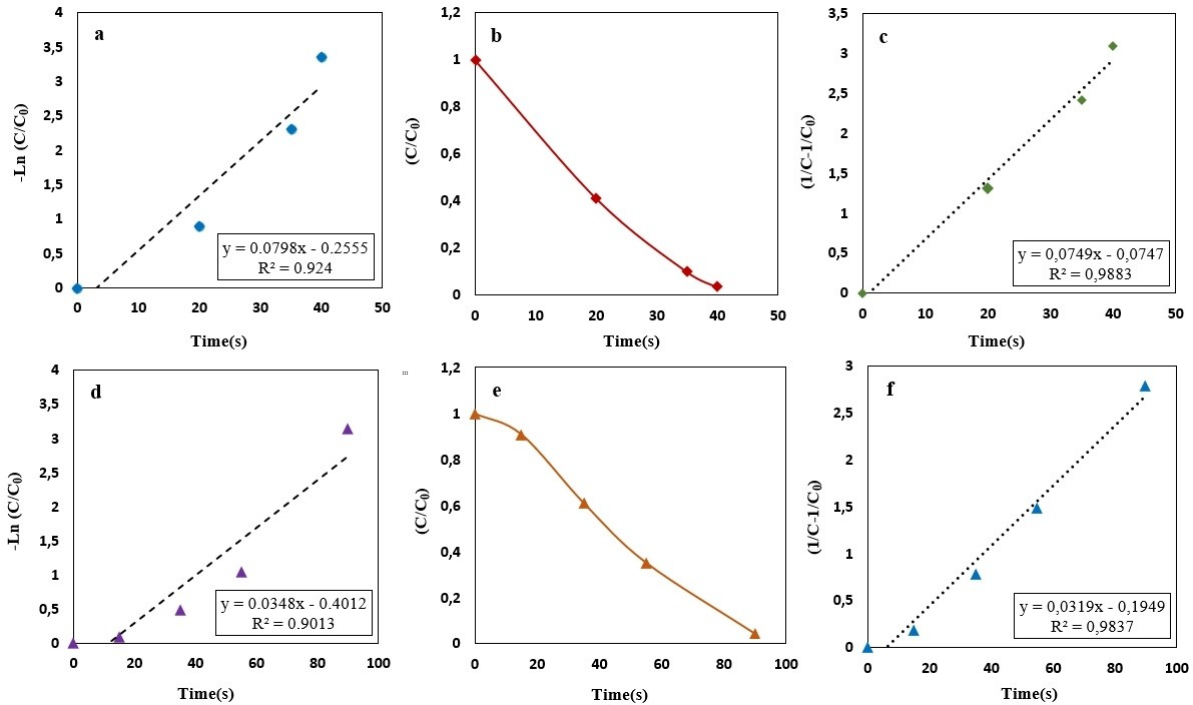
Plots of −ln(C_t_/C_0_) vs. time for the reduction of 4‐NA to *para*‐PDA (a, b, c) and 2‐NA to *ortho*‐PDA (e, d, f) with nano‐catalyst at room temperature (25 °C) using NaBH_4_ as hydrogen source (reducing agent).

Where $C_{t}$ and $C_{0}$ demonstrate the nitroarenes′ concentration or absorbance at t=0 and t at any time, respectively. $K_{app}$ is the apparent constant rate for synthesized nano‐catalyst. To have a comprehensive quantitative catalytic rate of the CuFe_2_O_4_ NPs‐catalyzed nitroarene reduction, illustrated in Figure 10, the values were calculated and listed in Table 1. Figure 10(b, e) exhibits the concentration of substrate that decreased with time due to the decreasing the concentration of 4‐NA (b) and 2‐NA (d) and increasing the concentration of the new products, that is, *p*‐PDA and *o*‐PDA, respectively. The pseudo‐first‐order kinetic reaction equation illustrated an unfavorable quality of the linear fit in all nitroaniline samples as represented in Figure 10(a, d). In contrast, the data can be perfectly fitted by a pseudo‐second‐order kinetic reaction equation, which is shown below in Equation 7.

(7) $$\frac{1}{C}-\frac{1}{C_{0}}=K_{2}\cdot t$$

**Table 1:** Results of 4‐NA and 2‐NA reduction in the presence of the nano‐catalyst.

Nitroaromatics	Conversion [%]	R^2^	*K*_*app*_ [s^−1^]	Completion time [s]
4‐NA	96.5	0.988	7.49×10^−2^	40
2‐NA	95.6	0.983	3.19×10^−2^	90

where $K_{2}$ (L mg^−1^ min^−1^) is the second‐order kinetics constant rate, and was determined from a linear fit to the data.^[52]^ The obtained results using pseudo‐second‐order kinetic reaction equation are presented in Figure 10(c, f), showing that they had a linear behavior using this equation.

To examine the performance of synthesized CuFe_2_O_4_ NPs, we enumerated them from the $k{}^{'}=K_{app}/m$ formula. Where $k{}^{'}$ is the ratio of the constant rate $K_{app}$ (s or min) to the amount of the nano‐catalyst m (mg), which was loaded into the solution to reduce nitroaromatic compound's reaction. Given the formula, the value of the nano‐catalyst ($k{}^{'}$ ) for 4‐NA and 2‐NA is about 0.0798 s^−1^/3.5 mg or 0.0228 s^−1^ mg^−1^ and 0.0348 s^−1^/3.5 mg or 0.0099 s^−1^ mg^−1^, respectively.^[53]^

### Comparison of the obtained CuFe_2_O_4_ NPs catalyzed 4‐NA and 2‐NA with recent studies

To demonstrate the remarkable outcome of this study, the results were compared with recently conducted investigations (Table 2). The comparative table shows that the currently used CuFe_2_O_4_ NPs as heterogeneous catalysts are cost‐effective, environmentally friendly, and profitable. It is much better than the previously reported catalysts, which have been reported as a promising candidate for catalyzing the 4‐NA and 2‐NA in terms of catalyst dose, time, rate, and reduction percentage.

**Table 2:** A summarized literature review of recently conducted research for reduction of 4‐NA and 2‐NA.

Catalyst	Catalyst amount	*K_app_* [s^−1^]	Recycling runs	Temperature [°C]	Reducing agent	Reducer concentration	Time	Nitroaromatic	Ref.
Ni/RGO	10 mg/50 mL	1.29×10^−2^	–	30	NaBH_4_	0.5 g/20 mL	190 min	4‐NA	[27]
Ag‐PNiM hybrid microgels	4 mg mL^−1^	8.52×10^−1^	–	25	NaBH_4_	32.15 mm	13 min	4‐NA	[54]
ZnO/CdO/RGO	1.2 mg L^−1^ in 25 mL	7.1×10^−3^	4	25	–	–	120 min	4‐NA	[55]
ZnO NPs	10 mg L^−1^	2.44×10^−2^	–	25	–	–	105 min	4‐NA	[56]
CuFe_2_O_4_	3.5 mg/5 mL	7.98×10^−2^	6	25	NaBH_4_	100 mg/5 mL	40 s	4‐NA	This study
Pd/CoFe_2_O_4_/chitosan	4 mg	–	5	25	NaBH_4_	0.1 mL, 2.5×10^−2^ m	65 s	2‐NA	[10]
Au/SiO_2_‐shell/Fe_3_O_4_‐core	3 mg	4.1 ×10^−3^	10	30	NaBH_4_	1 mL (0.2 m)	4.2 min	2‐NA	[29]
CoMn_2_O_4_/APTPOSS@FPS	1 mg/2.5 mL	1.83×10^−2^	10	25	NaBH_4_	0.5 m	100 s	2‐NA	[50]
20 %V dopped‐Bi_2_ (O, S)_3_	10 mg	34.4×10^−3^	3	25	NaBH_4_	0.1 m, 5 mL	150 s	2‐NA	[53]
SiO_2_@CuxO@TiO_2_	10 mg	0.018	–	25	NaBH_4_	–	150 s	2‐NA	[57]
Ag NPs	0.50 mL	2.43 ×10^−3^	–	25	NaBH_4_	0.05 m, 1.00 mL	540 s	2‐NA	[58]
CuFe_2_O_4_	3.5 mg/5 mL	3.48×10^−2^	6	25	NaBH_4_	100 mg/5 mL	90 s	2‐NA	This study

### Investigating the reusability of nano‐catalyst

**Figure 11:**
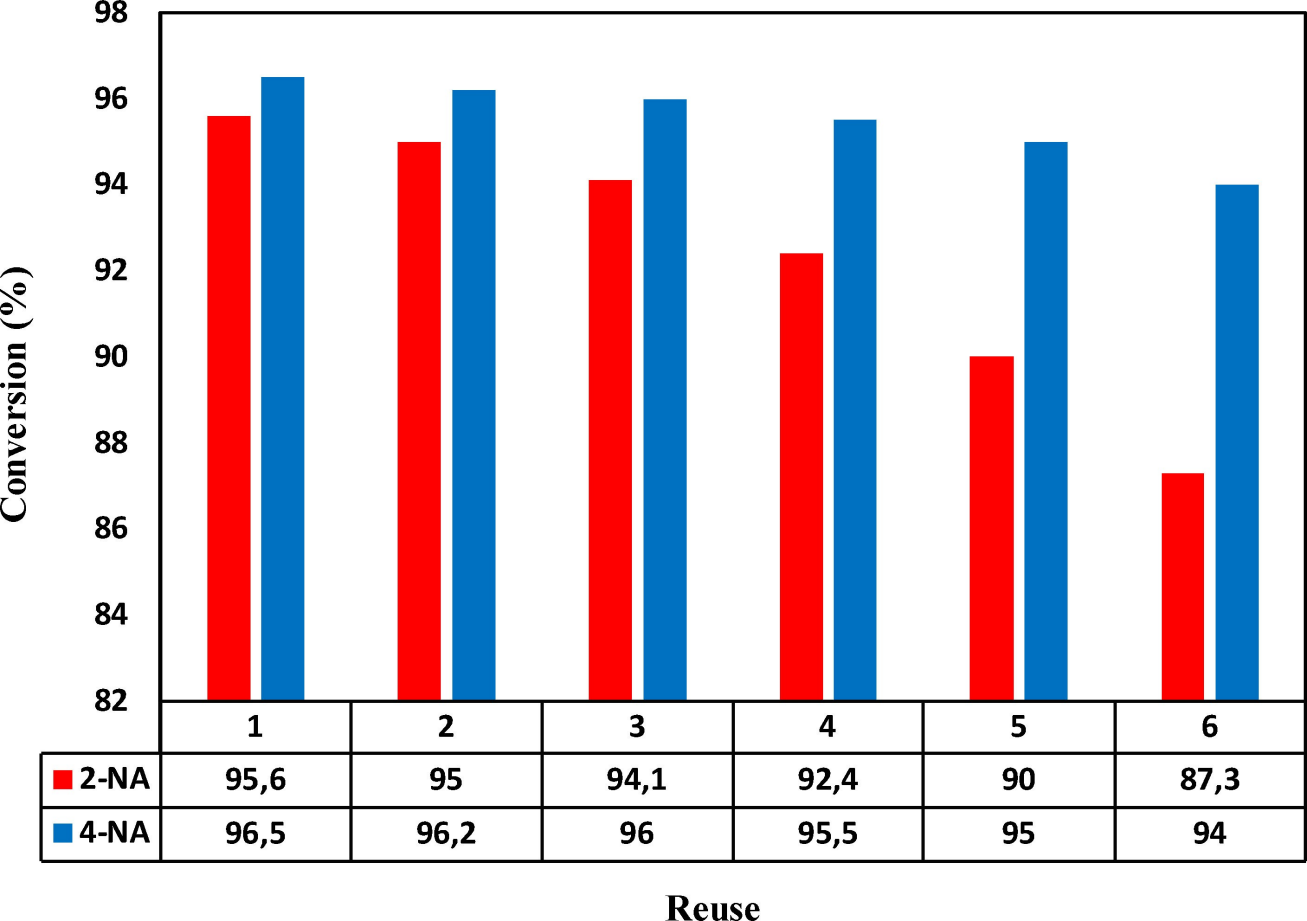
Conversion rate of the nano‐catalysts during six cycles.

To further investigate the synthesized nano‐catalyst's stability and reusability, the nano‐catalyst was recycled for several cycles and its stability and performance examined for up to six cycles. For this purpose, the catalyst was washed with water and ethanol after each use, and the reaction was completed, then dried by a pump. The synthesized nano‐catalyst performed well based on the reaction time mentioned to reduce the 4‐NA and 2‐NA, as shown in Figure 11. Accordingly, during the recycling, the conversion rate decreased from 96.5 % to 93.2 % for 4‐NA and 95.6 % to 86 % for 2‐NA, proving high stability and usability of the obtained nano‐catalyst.

## Conclusions

Magnetic CuFe_2_O_4_ NPs were synthesized as an effective, economical, and environmentally friendly nano‐catalyst for reducing 4‐NA and 2‐NA using sodium borohydride as reducing agent. The reduction product of 4‐NA and 2‐NA were *para*‐phenylenediamine (*p*‐PDA) and *ortho*‐phenylenediamine (*o*‐PDA). The constants rate (*k′*) for 4‐NA and 2‐NA were about 0.0228 s^−1^ mg^−1^ and 0.0099 s^−1^ mg^−1^. Comparing the obtained results with previously conducted works, it was found that the prepared nano‐catalysts had remarkable characteristics compared to the others. Also, the outcome demonstrated that the magnetic NPs as a nano‐catalyst had a high reusability characteristic in that the obtained catalyst could be used in reaction up to six cycles without any losses. The conversion rate decreased from 96.5 % to 93.2 % for 4‐NA and 95.6 % to 86 % for 2‐NA.

## Author contribution

**Samin Naghash‐Hamed**: Investigation, Conceptualization, Methodology, Formal analysis, Writing original draft. **Nasser Arsalani**: Supervision. **Seyed Borhan Mousavi**: Formal analysis, Writing original draft.

## Conflict of interest

The authors declare no conflict of interest.

1

## Data Availability

Data sharing is not applicable to this article as no new data were created or analyzed in this study.
